# Use of a Thermophile Desiccation-Tolerant Cyanobacterial Culture and Os Redox Polymer for the Preparation of Photocurrent Producing Anodes

**DOI:** 10.3389/fbioe.2020.00900

**Published:** 2020-08-21

**Authors:** Manuel Gacitua, Catalina Urrejola, Javiera Carrasco, Rafael Vicuña, Benjamín M. Srain, Silvio Pantoja-Gutiérrez, Donal Leech, Riccarda Antiochia, Federico Tasca

**Affiliations:** ^1^Departamento de Química de los Materiales, Facultad de Quiìmica y Biologiìa, Universidad de Santiago de Chile, Santiago, Chile; ^2^Departamento Genética Molecular y Microbiología, Facultad Ciencias Biológicas, Pontificia Universidad Católica de Chile, Santiago, Chile; ^3^Departamento de Oceanografía and Centro de Investigación Oceanográfica COPAS Sur-Austral, Universidad de Concepción, Concepción, Chile; ^4^School of Chemistry and Ryan Institute, National University of Ireland Galway, Galway, Ireland; ^5^Department of Chemistry and Drug Technologies, Sapienza University of Rome, Rome, Italy

**Keywords:** *Gloeocapsopsis* sp., cyanobacteria, photo-bioelectrochemistry, photosynthesis, osmium redox polymer

## Abstract

Oxygenic photosynthesis conducted by cyanobacteria has dramatically transformed the geochemistry of our planet. These organisms have colonized most habitats, including extreme environments such as the driest warm desert on Earth: the Atacama Desert. In particular, cyanobacteria highly tolerant to desiccation are of particular interest for clean energy production. These microorganisms are promising candidates for designing bioelectrodes for photocurrent generation owing to their ability to perform oxygenic photosynthesis and to withstand long periods of desiccation. Here, we present bioelectrochemical assays in which graphite electrodes were modified with the extremophile cyanobacterium *Gloeocapsopsis* sp. UTEXB3054 for photocurrent generation. Optimum working conditions for photocurrent generation were determined by modifying directly graphite electrode with the cyanobacterial culture (direct electron transfer), as well as using an Os polymer redox mediator (mediated electron transfer). Besides showing outstanding photocurrent production for *Gloeocapsopsis* sp. UTEXB3054, both in direct and mediated electron transfer, our results provide new insights into the metabolic basis of photocurrent generation and the potential applications of such an assisted bioelectrochemical system in a worldwide scenario in which clean energies are imperative for sustainable development.

## Introduction

**SCHEME 1:**
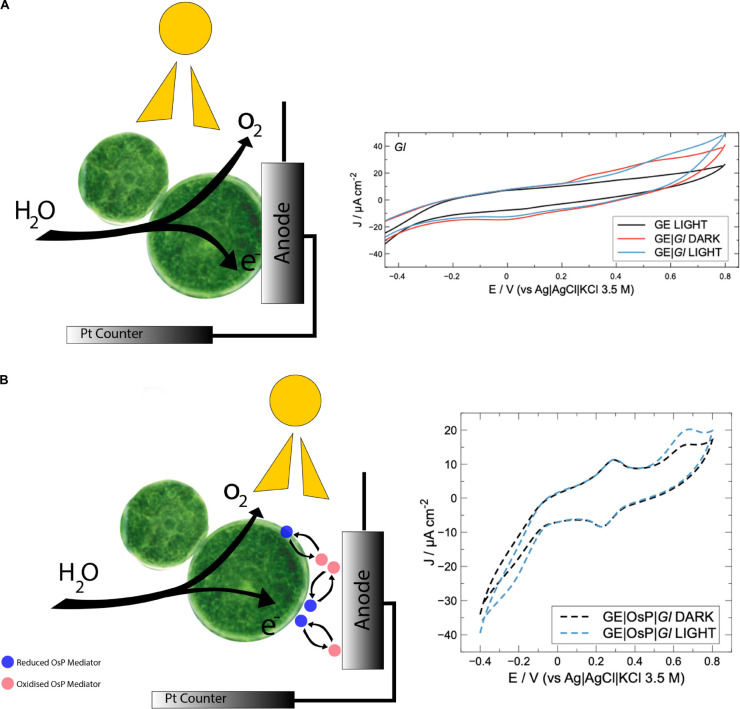
**(A)** Schematic modification of graphite electrode with Cyanobacteria. Under light conditions, e^–^ and O_2_ are produced and water is oxidized. Electrons can reach directly the electrode. During cyclic voltammetry experiments with *Gloeocapsopsis* sp. UTEXB3054, a positive current can be noticed in correspondence of an anodic redox peak (0.5 V). **(B)** Schematic modification of graphite electrode with cyanobacteria and Os polymer. Under light conditions, e^–^ and O_2_ are produced and water is oxidized. Electrons can reach the electrode surface through Os polymer redox centers which are in high concentration and can penetrate cyanobacteria membranes. During cyclic voltammetry experiments with electrode modified with *Gloeocapsopsis* sp. UTEXB3054 and Os polymer, a positive current with onset at 0.5 V can be noticed in correspondence of an anodic redox peak (0.6 V). Os redox peak at 0.24 V.

The environmental consequences of the current dependency of worldwide economy on fossil fuels have brought attention to alternative energy sources, among which solar energy-based photovoltaics represent the most appealing one. The solar radiation might also be used as energy source when assisted with photosynthetic organisms, forming photo-bioelectrochemical cells (Deng and Coleman, 1999; McCormick et al., 2011; Kaiser et al., 2013). These bioelectrochemical cells are based on the wiring of whole photosynthetic organisms or parts of their photosynthetic machinery to electrodes (Zhang et al., 2018), attempting to efficiently convert sunlight energy into electrical energy (Rosenbaum et al., 2005; Pankratova et al., 2017, 2019a; Pankratova and Gorton, 2017). The main principle of such technology is the interception of the electrons that flow from the water photolysis through the photosystem I and II (PSI and PSII). Cyanobacteria are exceptionally good candidates for assisting generation of photocurrent for three main reasons: (1) they generate electricity from their photosynthetic electron transport chain; (2) cultivation at industrial scale is possible and inexpensive; (3) feasibility of engineering the cyanobacterial genome for desired metabolic improvements. In cyanobacteria, photons are absorbed from the photosystems’ antennas and the energy is transferred to the PSII and PSI and used to drive electrons which are transferred from water to reduce NADP^+^. In the absence of light, cyanobacteria oxidize carbon sources via the respiratory system to consume oxygen and produce CO_2_ and ATP. Nevertheless, the exchange of electrons from the cyanobacterial electron transport chain to external electrodes is not a simple task. Aiming to optimize this process, cyanobacteria have been grown or dried on the top electrodie surfaces (McCormick et al., 2011; Cereda et al., 2014; Sekar et al., 2016; Wei et al., 2016). In other cases, they have been exposed to small pressures with a microfluidizer to obtain modification of the internal structure that allowed for endogenous mediators to shuttle electron between the respiratory chain and the PSI and to electrodes (Saper et al., 2018). The electron transfer from the inner side of cyanobacteria to electrode surface might be direct (where some proteins or endogenous mediator does provide for the electron transfer) or mediated by added biological or chemical redox mediators (Schemes 1A,B) (Torimura et al., 2001; Tsujimura et al., 2001; Yehezkeli et al., 2012; Longatte et al., 2017; Pankratova et al., 2019a, b). In particular, osmium complex-based redox polymers have been extensively studied in combination with various microorganisms showing an enhanced electron transfer between the electron cascades in microbial cells and the electrodes (Timur et al., 2007b; Rawson et al., 2011; Hasan et al., 2012, 2014, 2015; Hamidi et al., 2015; Pankratova et al., 2019b). Therefore, the preparation steps of an electrochemical system constituted by photosynthetic bacteria might imply that the bacteria have gone through an osmotic stress because of a desiccation step for the preparation of the modified electrodes and because of the need of redox mediators. Moreover, if bio-photovoltaic cells should be considered for major electricity production (e.g., home bio-photovoltaic systems), desiccation might also occur because of uncontrolled environment conditions and evaporation. A photosynthetic organism able to withstand extreme stress and long periods of dryness will therefore be a suitable candidate for testing photocurrent generation. *Gloeocapsopsis* sp. UTEXB3054 is a unicellular cyanobacterium originally isolated from Atacama, the driest warm desert on Earth, with an impressive ability to tolerate desiccation periods based on unique genetic features associated to desiccation tolerance (Urrejola et al., 2019). Because of these properties, its genome and sugar biosynthesis pathways have been studied (Urrejola et al., 2019, 2020). In this work, for the first time, we explored the use of *Gloeocapsopsis* sp. UTEXB3054 as part of a photo-bioelectrochemical system for testing photocurrent generation. Both direct and mediated electron transfer using a high-potential redox mediator were evaluated, as represented in Schemes 1A,B. Moreover, a biochemical characterization of *Gloeocapsopsis* behavior under desiccation was also conducted. Our results are discussed with relation to cellular strategies to deal with the lack of water and the implications that might affect green and sustainable technologies for energy generation.

## Experimental

### Chemicals

All the reagents for MLA growth medium and Lysogeny Broth medium, K_2_HPO_4_, KH_2_PO_4_, KOH, peptone, yeast extract, glucose, NaCl, fluorescein diacetate (FDA), methanol (MeOH), dichloromethane (DCM), hexane, acetone, HCl, BF_3_, and poly(ethyleneglycol) diglycidyl ether (PEGDGE) were from Sigma Aldrich (San Luis, MI, United States). The osmium polymer (OsP) [Os(bpy)_2_(poly vinylimidazole)_10_Cl]^2+/+^ was synthesized as previously described (Forster and Vos, 1990; Boland et al., 2008).

### Culture Preparation

*Escherichia coli* JM109 cell biomass (*Ec*) was grown by aerobic cultivation at 37°C in a 25-ml flask filled with 5 ml of medium for 72 h. The growth medium contained 10 g l^–1^ peptone, 5 g l^–1^ yeast extract, and 5 g l^–1^ NaCl. To prepare the *Ec* solution, 5 ml of the *Ec* suspension was mixed with 10 m0 of glucose water solution (5.55 × 10^–3^ mol L^–1^) and 185 ml of the phosphate buffer (1.1 g l^–1^ K_2_HPO_4_, 0.32 g l^–1^ KH_2_PO_4_, and 8.5 g l^–1^ NaCl, pH 7.4). *Gloeocapsopsis* sp. UTEXB3054 cyanobacterial cells (*Gl*) were grown using MLA medium (Bolch and Blackburn, 1996), at 27°C, without agitation under white light on a 12/12 h light/dark cycle for up to 2 weeks. In both cases, cells were recovered by centrifugation at 4,000 rpm, for 10 min at 20°C. Bacterial concentration was determined through cell count under light microscope using a Neubauer chamber. The cells concentrated in the pellet were washed in 10^–3^ M phosphate buffer solution (PBS), adjusted to pH 7.0 with a concentration of 1 g ml^–1^ (wet weight) for electrochemical measurements. Electrodes modified with *Ec* were prepared following a similar procedure. Sterility of all the employed material and chemicals was assured by standard sterilization procedures and the employment of autoclaves (Systec D-45).

### Cell Viability and Metabolic Activity Measurements

Cyanobacterial cell survival upon desiccation was measured during 4 weeks. Despite bioelectrode preparation requiring solely a couple hours of desiccation (depending on the methodology from few hours to 12 h of desiccation), 4-week desiccation period was estimated as appropriate, considering the possibility of scaling up this technology to prototype or industrial levels, which would require bigger electrodes and longer desiccation times, or unavailability of water for longer times. For survival measurements, 0.01 mg of scraped cells were rehydrated with 1.0 ml of pure water and immediately stained. Cell viability and metabolic activity were determined using the membrane-impermeable stain SYTOX Blue (Molecular Probes) and FDA (Sigma-Aldrich), according to the manufacturer’s instructions. This dye, which fluoresces on 408 nm excitation, has a high affinity to nucleic acid and only penetrates damaged cell membranes. The corresponding images were obtained using a Nikon Eclipse Ti inverted microscope, in spectral mode. The red chlorophyll fluorescence of live and dead cyanobacterial cells was excited with a 488-nm laser. Fluorescence was determined in technical replicates from more than four different microscopic fields of at least two different slides prepared under identical conditions. Photomicrographs were analyzed using ImageJ software^1^ (National Institutes of Health, Bethesda, MD, United States). On the other hand, FDA was dissolved in acetone in a concentration of 5 mg ml^–1^ and this stock was stored at −20°C. Starting from the stock solution, a 1:20 working solution in water was freshly prepared before each assay and maintained in darkness. Then 50 μl of this solution was added to 200 μl of culture, and incubated for 5 min under room temperature and darkness. To stop the reaction, samples were incubated for 5 min at 4°C. Fluorescence was determined in replicates from at least four different microscopic fields on each of the slides prepared under identical conditions. Stained cells were visualized using an Olympus FV 1000 confocal laser scanning microscope (Olympus, Hamburg, Germany) and images were recorded with the Fluoview software.

### Fatty Acid Extraction

Liquid cultures of *Gloeocapsopsis* sp. UTEXB3054 during active growth were harvested at room temperature by centrifugation (3,000 rpm) and subsequently lyophilized. All samples were maintained frozen at −80°C until extraction. To obtain enough biomass of desiccated samples, the organic extractions were performed with more than 10 independent desiccation experiments each. Three independent extraction experiments were performed using samples from independent desiccation experiments. Total lipid extracts were obtained from samples according to a modified method of Bligh and Dyer (Bligh and Dyer, 1959). Briefly, samples were sequentially extracted by ultrasonication for 30 min with 25 ml DCM/MeOH (1:1 v/v, 1×) and DCM (1×). Both DCM and MeOH caused the mixture to separate into organic and aqueous phases, from which the organic fraction was collected. The latter was then mixed with water and placed at 4°C for 12 h. Once two phases were obtained, the organic one was recovered and evaporated at 40°C in *rotovap* (Turbo Vap LV concentration workstation). Fatty acids (FAs) were obtained from the total lipid fraction by acid hydrolysis after saponification of the samples. Samples were resuspended in 2 ml of hexane and saponified in 15 ml of KOH/MeOH 0.5 N (Christie, 1989) at 80°C for 2 h. Further, 20 ml of hexane was added before sonication for 10 min. This recovery–saponification cycle was repeated two more times. The alkaline phase was then acidified with 3 ml of HCl 6 N. To finally obtain the FAs, the following cycle was repeated three times: addition of 20 ml of hexane and sonication for 10 min. The organic phase was then dried in *rotovap* at 40°C and totally recovered in 1 ml of hexane. Finally, samples were dried under nitrogen flux. FA methyl esters were obtained by heating with 1 ml BF_3_/MeOH (10%) at 70°C for 1 h. Final extracts were dried in *rotovap* and then reconstituted in 0.5 ml of hexane. FA methyl esters extracts were analyzed by gas chromatography coupled to mass spectrometry (GC-MS) using an Agilent 6890 GC series coupled to Agilent 5972 mass spectrometer, equipped with a HP5-MS column (30 m × 0.25 mm × 0.25 μm), using He as carrier gas. The GC oven program was 80°C (2 min) to 120°C at 20°C min^–1^, and then ramped at 4°C min^–1^ into 290°C. The MS was operated in electron impact mode (70 eV) with the ion source at 230°C. FAs methyl esters were identified by both the retention times and comparing the obtained mass spectra with reference mass spectra using the NIST MS search program. FA methyl esters are named here in the form of C:D, where C indicates the number of carbons and D the number of double bonds in the FA molecule.

### Electrode Preparation and Bioelectrochemical Experiments

**FIGURE 1:**
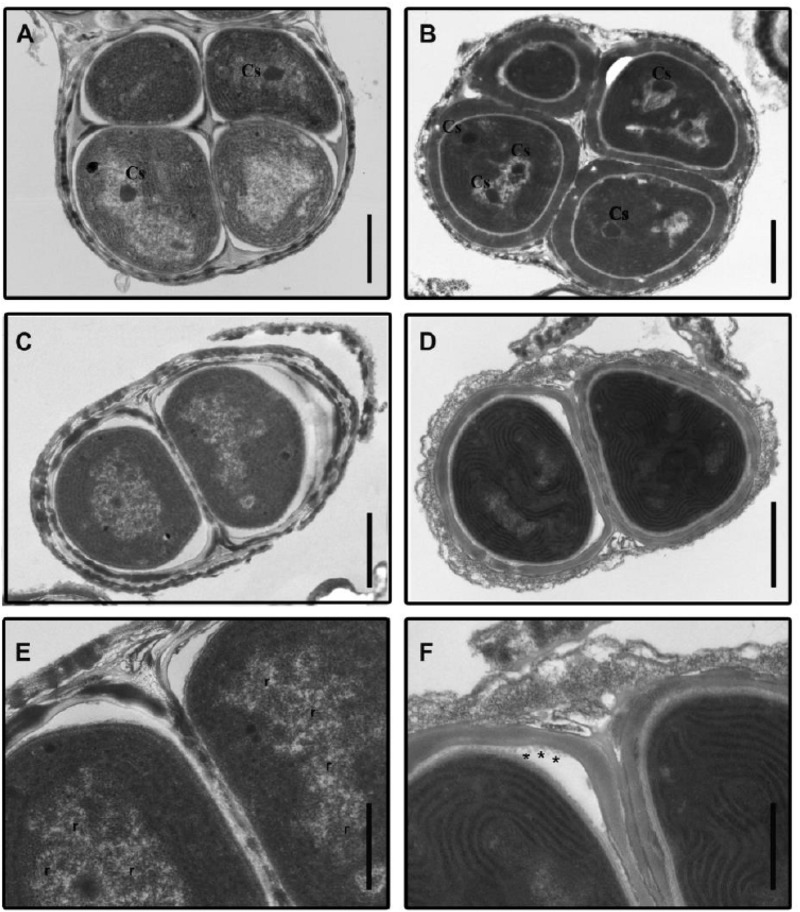
Transmission electron micrographs of *Gloeocapsopsis* sp. UTEXB3054 cells before and after desiccation. **(A)** Characteristic tetrad from a liquid culture, before desiccation (initial condition). Cs, carboxysome. Scale bar: 1.0 μm. **(B)** Characteristic tetrad after 3 weeks of desiccation. Scale bar: 1.0 μm. **(C)** A binary group of cells at the initial condition. Scale bar: 1.0 μm. **(D)** A binary group of cells after 4 weeks of desiccation. Scale bar: 1.0 μm. **(E)** Photomicrograph showing details of **(C)**. The thylakoid membranes are arranged in parallel in the cell periphery. r: presence of ribosomes. Scale bar: 0.5 μm. **(F)** Photomicrograph showing details of **(D)**. Asterisks indicate the presence of vesicles 0.5 μm.

**FIGURE 2:**
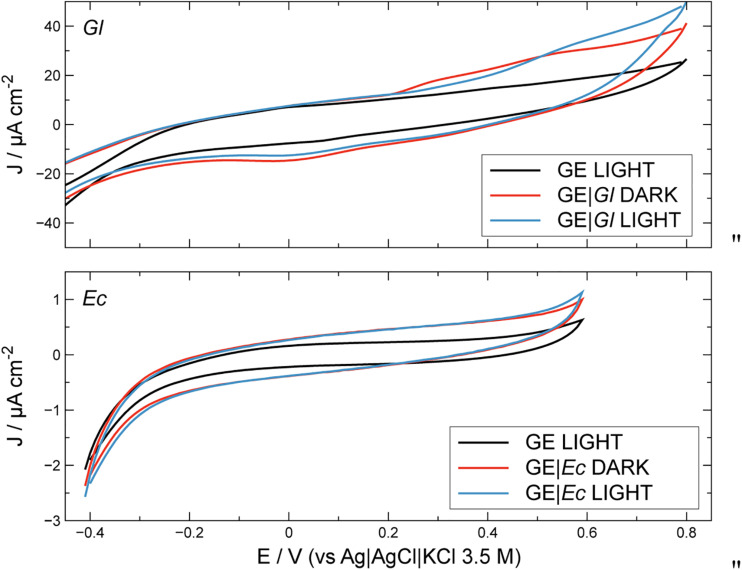
Cyclic voltammetry profiles for unmodified and modified electrodes, in the presence and in the absence of light. Conditions: PBS 0.1 M at pH 7.0. Scan rate: 10 mV s^–1^. GE, graphite electrode; Gl, *Gloeocapsopsis* sp. modification; Ec, *Escherichia coli* modification.

**FIGURE 3:**
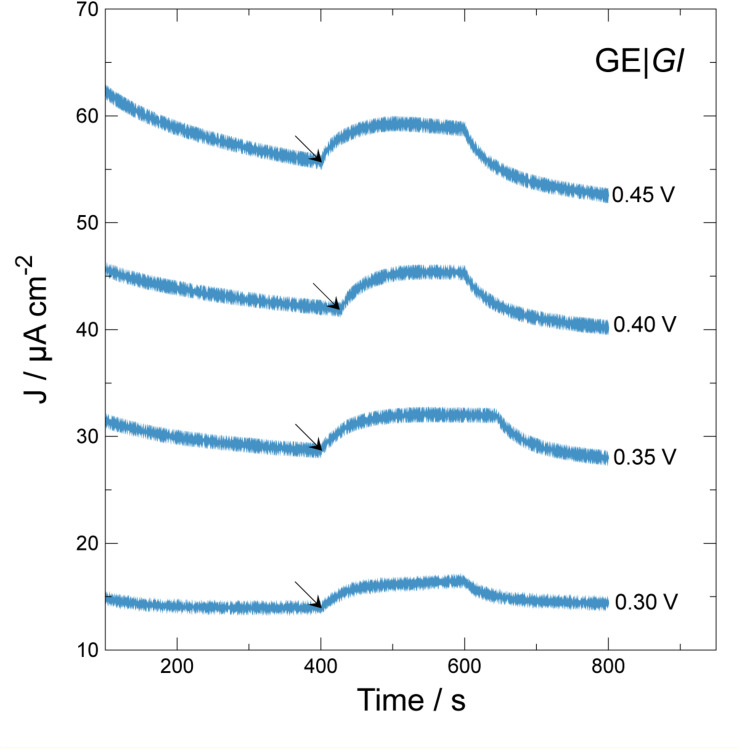
Effect of applied potential vs. current density generation over time during chronoamperometric test using GE| *Gl*. Arrows indicate beginning of illumination step for 200 s. Applied potentials: 0.30 V; 0.35 V; 0.40 V; 0.45 V vs. Ag| AgCl| KCl 3.5 M.

**FIGURE 4:**
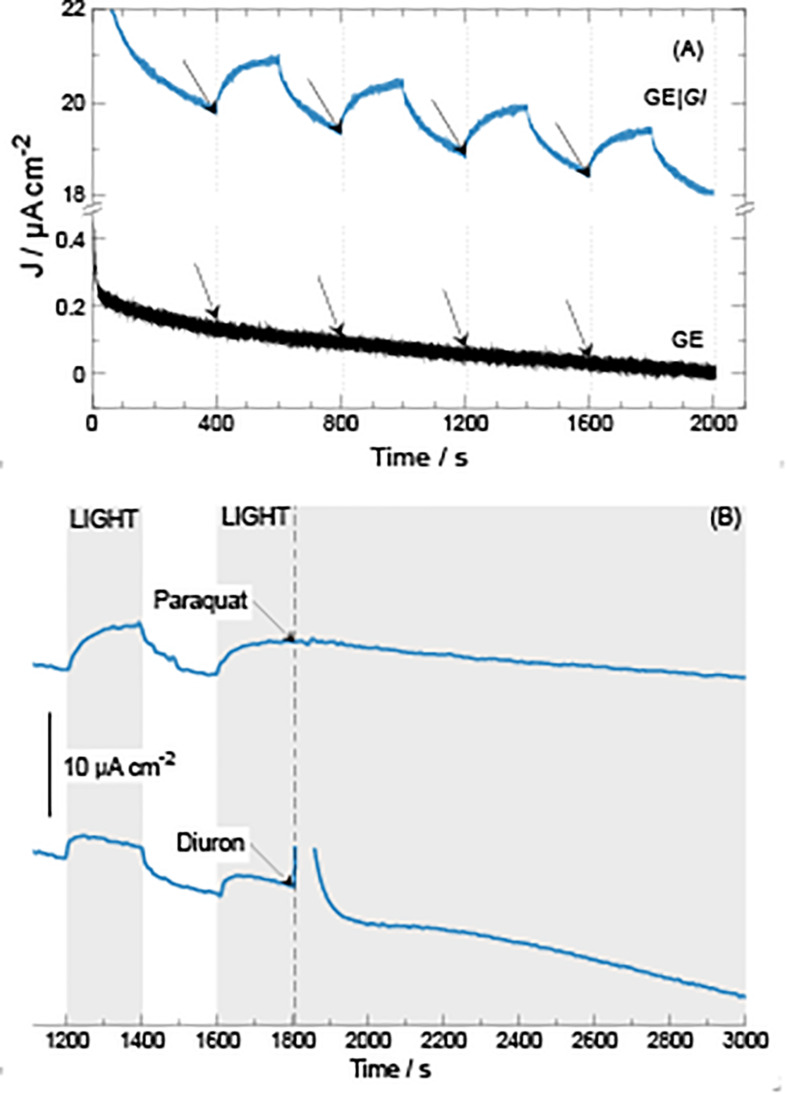
Current density generation over time during chronoamperometric tests at 0.40 V vs Ag| AgCl| KCl 3.5 M at GE| *Gl* electrodes. **(A)** Application of consecutive 200-s illumination steps. **(B)** Application of 200-s illumination steps followed by addition of either paraquat or diuron (20 μmol) at 1,800 s.

**FIGURE 5:**
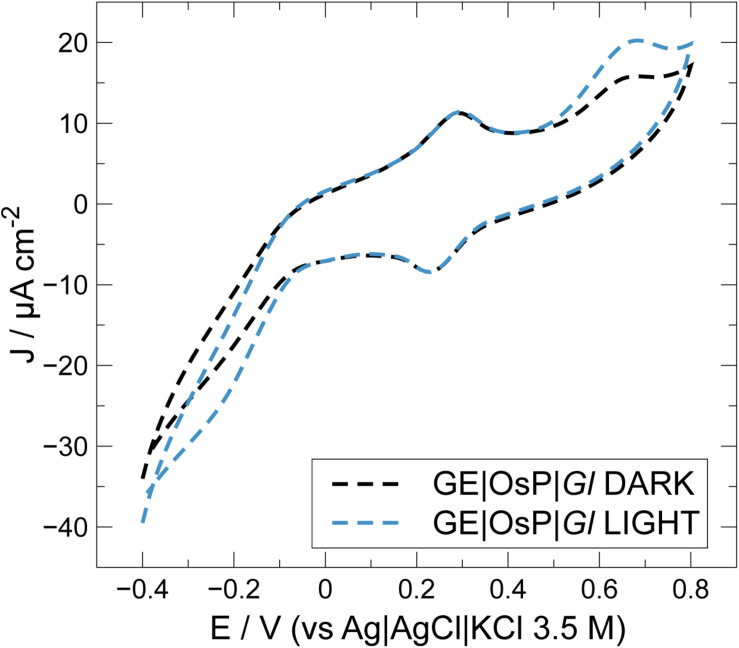
Effect of OsP on current density outputs during cyclic voltammetry experiments at electrodes modified with GE| OsP| *Gl*.

All electrochemical experiments were performed in K_2_HPO_4_, KH_2_PO_4_ (phosphate buffer) at pH 7.0. Edge-plane pyrolytic graphite electrodes (GE) of 5 mm diameter were from PINE (Durham, NC, United States). GE have been employed extensively in bioelectrochemistry because of the relatively smooth surface area and the good compatibility with proteins and microorganisms (Zafar et al., 2009; Tasca et al., 2015; Venegas et al., 2017). Before modification, the electrode surfaces were polished with emery paper (P800 and P1200) and carefully washed with distilled water and by sonication for 15 min in 95% ethanol to ensure sterility. Subsequently, the electrodes were left to dry at room temperature inside a laminar flow hood. Once the electrode was dry, direct electron transfer (DET) was estimated after adding 5 μl of PEGDGE (1 mg ml^–1^ solution) and 20 μl of resuspended cyanobacteria on the electrode surface (cultures were prepared to final bacterial concentration of 2.3 × 10^6^ cells ml^–1^). PEGDGE was introduced because of its ability to create hydrogel by crosslinking that prevents proteins and bacteria from desorption from the electrode surface (Zafar et al., 2009; McCormick et al., 2011; Cereda et al., 2014; Tasca et al., 2015; Sekar et al., 2016; Wei et al., 2016). For mediated electron transfer measurements, 10 μl of OsP (10 mg ml^–1^) was included over the electrode. A three-electrode arrangement was employed considering (1) an Ag| AgCl (3.5 M KCl) as reference electrode, (2) GE with a surface area of 0.196 cm^2^ as working electrode, and (3) a high surface area platinum counter electrode. GEs modified with *Gl* (GE| *Gl*), with *Gl* and OsP (GE| OsP| *Gl*), or with *Ec* (GE| *Ec*), or *Ec* and (OsP GE| OsP| *Ec*) were dried overnight at room temperature before being employed. Potentiodynamic (cyclic voltammetry) and potentiostatic (chronoamperometry) methods were employed with different conditions depending on the measurement. All experiments were conducted at room temperature and were repeated at least four times. Representative experiments are reported in Figures 1–5, whereas in Table 2, the average value for *J* with the corresponding SD are reported. During photo-bioelectrochemical experiments, a flexible lamp illuminated the working electrode surface with a light intensity of 44 mW cm^–2^. This light allowed excitation of the photosynthetic activity of *Gl*. All electrochemical measurements were carried out using a PalmSens potentiostat equipped with the PSTrace software for instrument control and data acquisition. ANOVA performed on the produced photocurrent densities (Δ*J*) for GE| *Gl*, GE| OsP| *Gl*, and GE| *Ec*, GE| OsP| *Ec* (Table 2) showed that there were effective statistical differences (95% confidence) between the various electrode modifications.

### Statistical Analysis

ANOVA was performed to analyze the differences between the photocurrent densities (Δ*J*) using Excel software.

## Results and Discussion

### Culture Characterization and Survival on Desiccation Experiments

To generate photocurrent by using *Gloeocapsopsis* sp. UTEXB3054 as photo-electrocatalyst, cell cultures must survive desiccation conditions when placed over the electrodes. As a first approach, different staining techniques that could prove cell survival after desiccation were tested. The membrane-impermeable fluorescent dye from the SYTOX series, SYTOX Blue, has been extensively employed to determine the portion of dead cells in a wide spectrum of organisms (Wobus et al., 2003; Krause et al., 2007; Adav and Lee, 2008; Tashyreva et al., 2013; Roldán et al., 2014; Giannattasio et al., 2015; Mason-Osann et al., 2015). This stain is an organo-arsenical compound with high affinity to nucleic acids that easily penetrates cells with compromised membranes (Park et al., 2011). In agreement with results previously reported (Azua-Bustos et al., 2014), after desiccating the cyanobacterial cultures for 4 weeks, 94% of the population appeared to be alive (total cells = 970) using membrane integrity as criterion of viability. These results were further complemented with FDA staining, a molecule with different chemical properties than those of SYTOX Blue. FDA is a non-fluorescent compound that is able to passively enter cells owing to its hydrophobicity. However, once inside the cell, non-specific esterases break down the molecule into fluorescein, a highly fluorescent and polar molecule that remains in the cytoplasm (Dorsey et al., 1989). The dye has been associated to metabolic vigor, allowing the discernment between cells that are still metabolically active from the inactive ones. Before desiccation, 86% of cells tested positive for the FDA (FDA+) method. After 4 weeks of desiccation, the percentage of FDA + cells fall to only 15%, a situation that became reverted once rehydration of the culture occurred, increasing FDA + cells to 50% of the whole population within 1 h (a proportion that was maintained during the following hours). After rehydration, desiccated cells were able to colonize both liquid and solid BG11 media, a clear demonstration of metabolism reactivation.

Bioelectrochemical systems for current generation are based on electron movement in biological membranes. All the biological elements associated with current generation in cyanobacteria (photosynthetic elements) are located in biological membranes. Although several ultrastructural changes became evident throughout the desiccation process that could be linked to the high tolerance to water deprivation of *Gl*, the physical integrity of membranes was maintained as evident from Figure 1.

Because of the crucial role FAs play in the maintenance of membrane fluidity (and therefore, functionality), the FA profiles during desiccation were also explored (Table 1). GC-MS results indicated that most FAs detected are C16 and C18 in almost equal amounts, both accounting for 97% of all FAs. FAs of the C14, C15, and C17 series were detected in trace or minor levels (each below 1% of the total FA content). Therefore, we focused exclusively on FAs with 16 and 18 carbons, considering them all together as a whole (Table 1). Interestingly, the ratio of unsaturated to saturated FAs remained unaltered under desiccation conditions. No significant differences were detected between the control samples (culture at active growth in liquid medium) and the desiccated samples for up to 4 weeks (*p* > 0.05; non-parametric data were tested by Kruskal–Wallis ANOVA test). Desiccation appears not to affect the FA composition of *Gl*. Considering the ability of *Gl* to thrive under desiccation periods preserving membranes integrity and composition of FA, we hypothesized that *Gloeocapsopsis* sp. UTEXB3054 might be a good candidate for biolectrode constructions.

**TABLE 1:** Percentage content of fatty acid of *Gloeocapsopsis* sp. UTEXB3054 during desiccation.

Fatty acids	Liquid culture before desiccation	After 4 weeks of desiccation
Monoenoic	65.6 ± 0.4	59.5 ± 6.5
Dienoic	11.6 ± 1.6	9.36 ± 3.9
C16	49.6 ± 0.8	50.3 ± 1.9
C18	50.4 ± 0.8	49.7 ± 1.9
Unsaturated/total	0.8 ± 0.02	0.7 ± 0.10

*The values represent average percentages of three FA extractions ± SDs.*

**TABLE 2:** Effect of electrode modification on photocurrent density outputs during chronoamperometric tests with electrodes polarized at 0.4 V vs Ag| AgCl| KCl 3.5 M.

Electrode architecture	*J*_min_	*J*_max_	Δ*J*
	
	(μA cm^–2^)		
GE| *Gl*	19.3 ± 0.6	20.2 ± 0.70	0.96 ± 0.08
GE| OsP| *Gl*	3.50 ± 0.10	5.70 ± 0.30	2.26 ± 0.28
GE| OsP| *Ec*	0.88 ± 0.07	0.95 ± 0.07	0.07 ± 0.02

*Values presented ± SDs. Averages and SDs were calculated from four different experiments.*

### Bioelectrochemical Experiments

Electrochemical activity of modified and unmodified GEs with photoautotrophic bacteria can be estimated along with the contribution from illumination (Figure 2). Bare GE does not demonstrate photoelectrochemical activity; similar results were obtained for electrodes modified with *E. coli* (GE| *Ec*) which were used as non-photosynthetic control. The latter profiles under illumination can be considered as the biotic blank. Under dark conditions, GE with *Gloeocapsopsis* (GE| *Gl*) reached *ca.* 38.3 ± 2.02 μA cm^–2^ at the positive working potentials, whereas GE| *Ec* gave only around 0.9 ± 0.02 μA cm^–2^. Under light conditions, the GE| *Gl* increased its current density to 47.1 ± 1.7 μA cm^–2^ at the positive potentials, whereas for GE| *Ec* the current density reached 1.1 ± 0.1 μA cm^–2^. To optimize GE| *Gl* photocurrent density production, the effect of working electrode potential was studied during potentiostatic (chronoamperometry) tests, as shown in Figure 3.

As expected, when electrodes are poised at higher electrode potential values, ranging from 0.30 to 0.45 V versus Ag| AgCl| KCl 3.5 M, higher current density outputs and baselines are produced. To select for optimal photocurrent density production, a single 200-s illumination step was applied, calculating photocurrent density difference (Δ*J*) between the beginning (see the arrows in Figure 3) and the maximum current density developed during the length of the step. When the system was polarized at 0.30, 0.35, 0.40, and 0.45 V, the Δ*J* values were 2.5 ± 0.2, 3.3 ± 0.0, 3.5 ± 0.3, and 3.3 ± 0.1 μA cm^–2^, respectively. These results indicate that GE| *Gl* at 0.40 V produces higher photocurrent density. Possibly, at values higher than 0.40 V, electrode components may become over-oxidized, losing its photoelectrochemical activity. For determining photocurrent density production, multiple illumination steps were applied monitoring current density (Figure 4A). Multiple illumination steps have no effect on GE; light does not alter current density output over time nor does it change the curve inflection (Figure 4A). In contrast, GE| *Gl* current density output undergoes an evident increase after each illumination step: an average 1.3 ± 0.1 μA cm^–2^ of Δ*J* increment owing to photocurrent density production can be calculated. Although we show only the first 2,000 s of the experiment, no decrease in current density could be noticed after 1 day of continuous cycles. To corroborate that the measured current density was generated by the photosynthetic cyanobacteria, two different photosystem inhibitors (i.e., diuron and paraquat) were included in working solution under continuous lighting for 1,200 s, after 1,800 s of continuous cycles (Figure 4B). Both inhibitors do not show strong light absorbance or interaction with the electrodes at this potential, or have the possibility to shuttle electrons (Hasan et al., 2014, 2017; Hamidi et al., 2015). Diuron is known to irreversibly block quinone A and B from photosystem II, hindering electron flow into plastoquinone (Rodea-Palomares et al., 2015; Rowen et al., 2017), whereas paraquat inhibition is known to take place at photosystem I, generating radical oxygen species (hydroxyl, superoxide, etc.) and finally damaging chloroplast structure and disrupting photocurrent density (Hupp and Meyer, 1989). Our results indicate that the generated current density is associated to electron flux of cyanobacterial photosynthesis (Figure 4B). Immediately after diuron addition (final concentration 0.2 mM), a current density peak was observed, but after ≈500 s, the inhibition of photosystem II took place. On the other hand, when paraquat is added (final concentration 0.2 mM) into the working solution, photocurrent density is inhibited after 900 s. Therefore, the measured current density is directly linked to the presence of photosynthetic organisms on the electrodes.

In an attempt to improve electrical wiring of electroactive bacteria, GE were modified with an OsP, an electrode wiring mediator previously used for wiring both enzymes and microorganisms to electrodes (Ohara et al., 1993; Vostiar et al., 2004; Timur et al., 2007a, b; Coman et al., 2009). Increased photocurrent outputs are usually obtained when in the presence of OsP because a higher number of molecules or living cells can be wired to the electrode because the OsP is usually present in excess and can penetrate the protein and membrane structures (Scheme 1B) (Kurbanoglu et al., 2018; Antiochia et al., 2019). In Figure 5, a typical CV obtained with electrodes modified with both OsP and *Gl* is presented. At 0.26 V, it is possible to observe the reversible peak for the OsP, corresponding to the Os(III)/Os(II) redox couple. Similar results were obtained in previous works (Hasan et al., 2014; Hamidi et al., 2015; Antiochia et al., 2013, 2019). Interestingly, at higher potential (0.65 V), a second oxidation peak is obtained. When in the presence of light, an anodic photo-electrocatalytic current appears with an onset at 0.45 V which can be assigned as related to the oxidation peak observed in dark conditions. Definitely the peak at 0.65 V belongs to one or more proteins wired to the photo-electrolysis system of *Gl*. In the presence of light, the Os(III) redox centers in the polymer matrix are reduced by available electrons occurring from the photo-electrolysis of the electrolyte. The formed Os(II) centers are acting as a relay of electrons and are then re-oxidized at the electrode surface (if the electrode is polarized at potentials positive enough for the oxidation to be consistent). When in the presence of light, at lower potential (−0.1 V), a cathodic current might be noticed which probably corresponds to the reduction of the oxygen produced by *Gl* during the photoelectrocatalysis.

A summary of photocurrent density production obtained with by GE| OsP| *Gl* during chronoamperometric measurements is presented on Table 2. OsP made a significant effect in the generation of photocurrent density (ANOVA test; *p* = 7.6 × 10^–7^). To compare the photocurrent generated by the two different electrode modifications used in this work, the background current has to be subtracted (Torimura et al., 2001; Tsujimura et al., 2001; Timur et al., 2007b; McCormick et al., 2011; Rawson et al., 2011; Hasan et al., 2012, 2014, 2015; Yehezkeli et al., 2012; Cereda et al., 2014; Hamidi et al., 2015; Sekar et al., 2016; Wei et al., 2016; Longatte et al., 2017; Pankratova et al., 2017; Pankratova and Gorton, 2017; Saper et al., 2018). In this case, the background current is consisting of the anodic current which is measured during dark chronoamperometric experiments in conditions (*J*_min_) and which is caused by the anodic polarizing potential. Therefore, the difference (Δ*J*) between the photocurrent measured during chronoamperometric experiments in light conditions (*J*_max_) and *J*_min_ must be analyzed (Table 2). The comparison of the *J*_min_ and *J*_max_ values for each type of electrode modification reveals that the OsP layer diminishes the background current density of the Gl bioelectrode. This type of behavior has been observed for other bioelectrode systems (Hasan et al., 2014; Hamidi et al., 2015). Once comparing photocurrent density productions, Δ*J*, a significant increase in photocurrent density production was observed: GE| OsP| *Gl* produces *ca.* 2.4 times more photocurrent density than GE| *Gl*, which was confirmed with statistical ANOVA with *p* < 0.05 (Table 2). The best-performing system obtained in this work corresponds to GEs modified with OsP and *Gl*, which yield a photocurrent density of 2.26 ± 0.28 μA cm^–2^ at 400 mV versus Ag| AgCl, KCl 3.5 M. OsP appears to be a good mediator to be used for light-energy to electrical-energy conversion because of its ability to accept electrons produced during the photosynthesis and shuttle them to the electrode. The photocurrent density production obtained in this work is within the range of photocurrent density production previously reported (Table 3).

**TABLE 3:** Comparison of photocurrent density output for various reports using whole living cells.

Organism	Electrode material	Redox mediator	Loaded biomass	Light intensity	Potential	Δ*J*	References
			(mg cm^–2^)	(mW cm^–2^)	(mV*)	(μA cm^–2^)	
*Paulschulzia pseudovolvox*	Graphite	OsP electrode phase	0.055	44	344	0.1	Hasan et al. (2015)
*Nostoc* sp. (cyanobacteria)	Carbon paper with carbon nanotubes	None	2.2	76	239	3.0	Sekar et al. (2014)
*Leptolyngbya* sp. (cyanobacteria)	Graphite	OsP electrode phase	0.13	44	244	4.5	Hasan et al. (2014)
*Gloeocapsopsis* sp. (cyanobacteria)	Graphite	OsP electrode-phase	0.1	44	400	2.3	Present work
		None		44	400	1.0	

*Versus Ag| AgCl, KCl 3.5 M.

## Conclusion

A new bio-photoelectrochemical system was studied, consisting on the modification of GEs with extremophile cyanobacteria *Gloeocapsopsis* sp. culture. During both the preparation steps of bio-photovoltaic cells and operations, the organisms responsible for the bio-photoelectrocatalytic currents might undergo osmotic stress and desiccation, hence our interest in extremophile cyanobacteria. Although *Gloeocapsopsis* entered into a metabolic arrest during desiccation, its biological membranes remained unaltered as mean of FA composition and viability tests, therefore becoming a good candidate for bioelectrode construction. Through cyclic voltammetry and chronoamperometric experiments, we determined that 0.40 V was the optimum potential for photocurrent density generation. The best-performing bioelectrode achieved 2.26 ± 0.28 μA cm^–2^ of current density when OsP was present as an electron mediator at the electrode surface.

## Data Availability Statement

The datasets generated for this study are available on request to the corresponding author.

## Author Contributions

MG, CU, and FT contributed with experimental design and writing of the manuscript. CU performed microscopy related experiments. CU and BS performed fatty acid related experiments. JC contributed running the electrochemical related experiments. RV, BS, SP-G, and RA contributed with experimental design and supporting with discussion and revision of the manuscript. DL contributed with the synthesis of the redox polymer and revision of the manuscript. All authors contributed to the article and approved the submitted version.

## Conflict of Interest

The authors declare that the research was conducted in the absence of any commercial or financial relationships that could be construed as a potential conflict of interest.

## Footnotes

1 http://rsb.info.nih.gov/ij/
